# Communication in youth mental health clinical encounters: Introducing the agential stance

**DOI:** 10.1177/09593543221095079

**Published:** 2022-06-22

**Authors:** Clara Bergen, Lisa Bortolotti, Katherine Tallent, Matthew Broome, Michael Larkin, Rachel Temple, Catherine Fadashe, Carmen Lee, Michele C. Lim, Rose McCabe

**Affiliations:** City University London; University of Birmingham; City University London; University of Birmingham; Aston University; McPin Foundation; McPin Foundation Young People’s Network; City University London

**Keywords:** empathy, epistemic injustice, sense of agency, validation, youth mental health

## Abstract

When young people seek support from mental health care practitioners, the encounters may affect the young people’s sense of self, and in particular undermine their sense of agency. For this study, an interdisciplinary team of academics and young people collaboratively analysed video-recorded encounters between young people and mental healthcare practitioners in emergency services. They identified five communication techniques that practitioners can use to avoid undermining the young person’s sense of agency in the clinical encounter. They conceptualise the use of those techniques as the adoption of an agential stance towards the young person. The agential stance consists of: (a) validating the young person’s experiences, (b) legitimising the young person’s choice to seek help, (c) refraining from objectifying the young person, (d) affirming the young person’s capacity to contribute to positive change, and (e) involving the young person in the decision-making process.

## Sense of agency in clinical encounters

In this article, we discuss communication techniques that can improve the quality of interactions between mental healthcare professionals and young people seeking the support of emergency services for a mental health crisis. We focus on how practitioners can adopt an agential stance towards young people and we discuss the potential benefits and risks of such a stance. Practitioners adopt the agential stance when they treat the young person as someone who has *agency*. Agency is a person’s capacity to intervene in their surrounding physical and social environment in order to pursue their goals and interests. In the context of a mental health crisis, goals may be health-related (e.g., improving one’s mental health). A person’s *sense of agency* is their perception of their own agency: a person who feels helpless typically does not have a strong sense of agency. A person who has just successfully fulfilled a long-term goal, overcoming difficulties, typically has a strong sense of agency.

For young people who struggle with their mental health, clinical encounters present a risk that their sense of agency will be undermined (e.g., Houlders et al., 2021). This risk may be higher than for other healthcare service users for four reasons. First, a young person’s identity is still developing and is more susceptible to external influences, especially in the context of social interactions characterised by power imbalances, such as the interaction between a practitioner who can offer support and a person who needs support.

Second, the sense of agency is moulded by the person’s closest social relationships: indeed, in some contexts it makes sense to talk about a person’s sense of *relational agency*, which points to the capacity a person has to influence and be influenced by significant others and contribute to shaping the relationship (De Mol et al., 2018). For young adults, the closest relationships with family members and peers are “in flux” and typically need to be renegotiated at their developmental stage (Boden-Stuart et al., 2021).

Third, negative stereotypes associated with young people (young people being described as “snowflakes” or “drama queens”) may influence the practitioner’s behaviour: unwarranted judgements about young people being immature, irresponsible, selfish, or attention seeking may lead to dismissing their reports of their experiences or delegitimising their concerns (see, e.g., Byrne et al., 2021).

Fourth, the sense of agency of people who struggle with their mental health may already be threatened by their adverse experiences. This generates a sense of helplessness which prevents them from feeling that they have the power to contribute to positive change. For instance, the psychological construct of *perceived control* can be helpful here, as it is associated with the motivation to implement change and the capacity to cope with adverse circumstances (see, e.g., Skinner, 1995). Perceived control encompasses the belief that a person can at least in part determine their mental states, affect changes in their surrounding environment, and achieve their goals. In a mental health crisis, perceived control is under threat.

Why is it important not to undermine a person’s sense of agency? In interactions with power imbalances, epistemic injustice may arise. *Epistemic injustice* occurs when the subordinate party is not regarded as a credible or reliable knower by the dominant party for reasons that are irrelevant to the subordinate party’s capacities as a knower and depend rather on negative stereotypes associated with the subordinate party’s identity (Fricker, 2007). As a result, the dominant party may enjoy excessive self-trust whereas the subordinate party comes to question their own self-trust (Jones, 2012). This situation further compromises the subordinate party’s capacity to produce and share knowledge: self doubt can translate into people becoming less forthcoming in describing their experiences or less willing to share their feelings and views (Fricker, 2007). Thus, epistemic injustice is not only morally problematic but causes relevant knowledge to become unavailable.

Young people seeking care from mental health emergency services often report feeling that their concerns are invalidated and minimised by healthcare practitioners. This leaves young people feeling misunderstood, guilty for seeking support, and deterred from future help seeking (Byrne et al., 2021). One problem is that young people may not be willing to share what they think and feel with mental healthcare practitioners for fear of experiencing lack of understanding. This makes it harder to identify the best support for them. Another problem is that young people may avoid seeking help altogether if they have had an unsuccessful encounter with a mental healthcare practitioner, and will then miss out on attaining further support—it has been consistently found that good therapeutic relationships predict good clinical outcomes (e.g., Birkhäuer et al., 2017).

We are interested in how communication can foster relationships where mental healthcare professionals treat young people as agents. We argue that such interactions may help prevent cases of epistemic injustice and create the conditions for young people to talk openly about what they experience. When young people are not treated as agents in these interactions, there is a risk that practitioners are unable to access information about young people that is useful to providing adequate support and that young people are deterred from seeking help when they face another crisis.

Next, we describe our methodology. In the rest of the paper, we describe five communication techniques that practitioners can use to protect the young person’s sense of agency in the clinical encounter: (a) validating the young person’s experiences, (b) legitimising the young person’s choice to seek help, (c) refraining from objectifying the young person or from preemptively labelling them, (d) affirming their capacity to contribute to change, and (e) involving them in the relevant decision-making processes. We discuss the implications of adopting such communication techniques in the clinical encounter: our findings can inform the training of mental healthcare practitioners and young people’s expectations in seeking support.

## Methodology and ethics

This is the first outcome of a team collaboration involving two groups of researchers: a group of six experts in philosophy, psychology, psychiatry, clinical communication, clinical practice, and public involvement in research (Interdisciplinary Academic Researchers, IAR); and a group of five young people (ages 17–25) with experience of receiving mental healthcare (Youth Lived Experience Researchers, YLER). YLER members had between 4 and 16 years of experience with professional mental health support for diagnoses including posttraumatic stress disorder, major depressive disorder, generalized anxiety disorder, autism spectrum, and emotional dysregulation. The YLER were recruited by a charity specialising in public involvement in research via social media advertising and emails to the organisation’s listserv of lived experience volunteers. Both groups collaboratively analysed clips from video-recorded psychosocial assessments between emergency department (ED) psychiatric liaison practitioners and young people (ages 18–25) presenting with self-harm or suicidal ideation.

### Video data

Recordings were collected from a larger mixed-methods study of psychosocial assessments for self-harm and suicidality (see Bergen & McCabe, 2021; Xanthopoulou et al., 2021, for further details on these data and the larger study). Clips from 19 psychosocial assessments with 18 patients (aged 18–25) and 11 practitioners were analysed. Psychiatric liaison practitioners were mental health nurses, junior doctors, social workers, and other professionals. Patient exclusion criteria were: cognitive difficulties, active psychosis, requiring an interpreter, or being subject to a restriction order. Patients presenting with suicidal ideation or self-harm were approached by a practitioner in the waiting room and assessed for capacity to give informed consent. A three-step consent process was developed with a panel of lived experience experts. Two GoPro cameras were placed in the assessment room and the assessment was recorded with no researcher present. Participants were recruited in England between September 2018 and April 2019. The ED psychosocial assessment involves an assessment of needs and risks, leading to a care plan involving recommendations for community-based services, referrals to outpatient care, or hospitalisation.

### Data analysis

An inductive, iterative approach to data analysis was adopted. A wide range of video clips potentially relevant to patient agency (e.g., treatment discussions, risk assessments, diagnostic assessments) were initially selected by a member of the IAR with specialisation in clinical communication and conversation analysis (see Sidnell & Stivers, 2012). Conversation analysis is a research methodology involving inductive micro-analysis of video-recorded interpersonal interactions. It often involves group analysis of the observable features of video-recorded interaction to identify potential patterns and communication practices of significance (see Stevanovic & Weiste, 2017).

The YLER met six times (12 hours total) to collaboratively analyse video data. Meetings with the YLER were facilitated by the IAR team member with specialisation in public involvement in research, who also had lived experience of receiving mental health care. Two members of the IAR with expertise in conversation analysis also participated and asked prompting questions. Prompting questions focused on (a) observable features of interaction, including verbal and nonverbal behaviors; (b) contextual factors and socially informed understandings of what was observed; and (c) the YLER’s perspectives on what they were observing and how it related to agency. These meetings were audio recorded and transcribed.

The IAR also met 24 times (24 hours total) to collaboratively analyse video data, with one member with expertise in conversation analysis asking prompting questions. YLER analysis was fed back to the IAR. The analysis informed the selection of subsequent video clips in an interactive research process. In turn, observations from the IAR informed selection of video clips and the agenda of YLER meetings. The IAR identified preliminary themes from the YLER and IAR data analysis sessions. These themes were then brought to the IAR for open discussion and further interpretation of meaning and significance.

### Formulating the agential stance

During meetings in which the YLER watched and analysed video data, the group discussed the concept of agency and what aspects of agency were most relevant to the clinical encounter. The YLER identified five aspects of agency that were important to them but were frequently seen to be undermined in the video-recorded psychosocial assessments: (a) an agent is a subject of experience and their perspective matters; (b) an agent can take action to change their situation by seeking help; (c) an agent may have multiple and conflicting needs and interests; (d) with adequate support, an agent can contribute to positive change; and (e) with adequate support, an agent can participate in decision making.

These five aspects of agency inform what we describe as the *agential stance*. Practitioners adopt the agential stance when they acknowledge that the young people are subjects of experience, with a perspective on the world that matters; communicate that the young people made the right choice in seeking support for their distress; recognise that the young people have a multiplicity of complex and potentially conflicting needs and interests that are partly shaped by their environment; affirm that, with adequate support, the young people can contribute to addressing the issues that cause them distress and make positive change; and involve them in the decision-making process about what support they need (see also Bellairs-Walsh et al., 2020). The aim of this work is to promote a reflection on what a good clinical encounter looks like and to inform future healthcare professional training.

## Validating the young person’s experience

### An agent is a subject of experience and their perspective matters

The YLER identified *validation* as a critical tool for demonstrating recognition that the young person’s experience and perspectives matter. Validation can be defined as a form of understanding and acceptance of another person’s internal experience, distinct from agreement or approval (Hall, 2011). Communication techniques contributing to validation of young people’s experiences in these data include attentive and empathetic listening, and accepting the young person’s experience at face value without immediately dismissing or challenging it. In the crisis care setting, mental health assessments often lack validation, as the primary focus is on risk assessment and problem solving. A lack of validation was perceived by the YLER as a cause of increased distress and poor rapport:

The problem. . . is that we all try to fix things rather than validate things. There’s no “oh that must be really bad for you,” it’s always “we need a solution to this problem right now.” And I think that’s where it all seems to go wrong. (YLER member)

By kind of bypassing those feelings it’s kind of like almost implicitly telling the person “okay your feelings are a problem, I’m uncomfortable with your feelings, we’re not going to talk about them we’re just going to fix them.” . . . It almost amplifies the young person’s distress. Because they’re struggling to sit with the feelings and they’re seeking help. (YLER member)

The YLER emphasised that, during a mental health crisis, good encounters with practitioners involve validation of the young person’s experiences, regardless of whether practitioners were able to identify solutions or next steps. Bad encounters with practitioners involve dismissing or contradicting the young person’s feelings or experiences.

In Extract 1, the practitioner listens to the young person’s description of their experience, but does not acknowledge the young person’s experience as an experience of distress:

Extract 1

1   Young person [YP]: I feel very unsafe in myself.

2  Practitioner [Pr]: Right.

3  YP: And that yeah I’m constantly having these horrible thoughts

4  and I just I’m in a lot of distress and there’s nowhere for

5  it to to go and

6  Pr: Right.

7  YP: Um

8  Pr: Okay.

…

50 YP: [crying] I can’t go on any longer like this. I just want to

51 be sedated. Just to wake up and for it all to go away.

52 Pr: Mm. Unfortunately that doesn’t happen does it. No.

53 YP: [sobs]

54 Pr: Alright then. Can we get you a glass of water or

55 anything like that?

The young person clearly describes their experience in lines 1–5. However, in lines 2 and 6, the practitioner responds minimally with no change in their tone of voice (“Right”) and no nonverbal feedback such as nodding or leaning in. After the second minimal response (“Right”), the young person stops describing their feelings midsentence. Disengagement and increased distress are typical responses to lack of validation in these data. Much later in the visit, the young person says they want to be sedated and for their feelings to go away, which the practitioner dismisses as unrealistic (line 52). Again, validation of the young person’s experience of distress is missing. The young person begins sobbing and they have to take a break from the assessment.

In contrast, the practitioner validates the young person’s distress in Extract 2:

Extract 2

1 YP: [crying] They died in a car crash.

2 Pr: Yeah. Yeah. [nods]

3 YP: So when- when I think about that it just fucks me up.

4 Pr: [nods] Of course. It’s a really horrendous event to have

5 happened isn’t it.

6 YP: Mhm. And obviously the other things are my ex that I had my

7 first child with um supposedly abused her basically. And I

8 heard that he’s been in the town where I live.

The young person tells the practitioner about a recent bereavement in line 1, then describes distress in line 3. Immediately, the practitioner nods, giving a nonverbal sign of affiliation and understanding (“It’s a really horrendous event to have happened isn’t it”). Although the reply is brief, the practitioner communicates that the young person’s feelings are understandable (“Of course”) and justified (“horrendous event”). After this, the young person opens up about another distressing experience—hearing that their ex-partner who may have abused their child has been seen in the town where they live. Across these data, young people seeking support routinely respond to validation by opening up and sharing more sensitive information.

A review of the full collection of video-recorded psychosocial assessments reveals that validation of young people’s experiences is rare. When there is validation, it is typically a response to the description of a traumatic event, as shown in Extract 2. The YLER highlighted the importance of also validating the young person’s subjective experiences of distress:

You need to say “You’re really distressed. You’re in a lot of pain.” I think that kind of acknowledgement alone can be really really powerful for someone who feels like they’re completely alone, isolated, and they don’t feel like they even have control over their own mind. (YLER member)

## Legitimising the young person’s choice to seek help

### An agent can take action to change their situation by seeking help

In general terms, *legitimisation* is the act of making something seem right, of justifying it. Legitimisation of help seeking expresses that the young person made the right choice in seeking help. Communication techniques contributing to the legitimisation of help seeking include clearly stating that the young person has a genuine concern, that they are deserving of support, and that they were right to take action and to ask for help.

An example of legitimisation is provided in Extract 3, where the practitioner legitimises the young person’s decision to attend the ED after self-harming and experiencing thoughts of suicide:

Extract 3

1 Pr: Okay? And it’s just about trying to get more to- to- You did

2 exactly the right thing today.

3 YP: [nods]

4 Pr: Exactly the right thing. So if you get to that point again,

5 you do exactly the same thing again.

6 YP: [big nod]

The young person’s decision to seek help is described as the right thing to do in the circumstances and they are encouraged to take the same action again in the future if they experience further thoughts of suicide.

In contrast, Extract 4 provides an example of a delegitimising approach:

Extract 4

1 Pr: So you had that talk [with the crisis team helpline] And

2 then you’ve come up to here. Did you discuss that with them

3 before you came up? Or-

4 YP: No. I rang the- I rang 111 first. And they said if you ring

5 the student health centre they’ll do an emergency telephone

6 thing. Then they said ring crisis team and if not go to A&E.

7 Pr: So what did you want them to do Anne. So I guess this is

8 what I’m struggling a little bit to get. What- What did you

9 think they could do?

The practitioner shows uncertainty over how crisis services could help, asking the young person to provide justification for seeking crisis support. Moreover, the young person is asked to identify what crisis services could do to help. The practitioner does not treat the young person’s decision to seek help as the right thing to do but rather implies that the young person’s distress does not deserve attention from the crisis team. Ultimately, responsibilities that should be held by the healthcare service are placed on the young person: identifying during a mental health crisis what support they need and where to seek that support. The YLER emphasised that identifying sources of support was not an area where they wanted to be handed more responsibility.

Though both young people in Extract 3 and Extract 4 presented to the service with thoughts of suicide, they were met with very different responses. Reflecting on the data we gathered, we can add that a delegitimising approach can take different forms and result from a number of considerations. Video analysis revealed four (nonexhaustive and nonexclusive) contexts in which practitioners fail to legitimise the young person’s decision to seek help: (a) the young person’s concerns are not regarded as genuine and so a question emerges as to whether there is need for support; (b) young people presenting themselves as knowledgeable or articulate may be told that their concerns are not sufficiently serious to justify support; (c) the young person is regarded as having other means of support available to them that could be relied on instead (e.g., the general practitioner, friends, the partner, or family members); and (d) the young person’s concerns are treated as genuine but the practitioner cannot identify suitable means of support.

Regardless of the reason, the YLER emphasised that failing to legitimise a young person’s decision to seek help can have a damaging effect:

With that sort of interaction, of “oh you’re not planning to do anything now so it’s fine,” you’re almost less likely to think “oh I’ve got these thoughts.” You know, the department’s said it’s not an issue so it’s fine sort of having those thoughts. . . . You wouldn’t think “well maybe it’s really serious maybe I do need to tell someone to help me” and you wouldn’t want to go seek the help again because of what happened previously. (YLER member)

When you’re really struggling with mental illness, there can be this really big overwhelming fear that your thoughts aren’t real or that you’re kind of overdramatising things or you’re being oversensitive or overreacting . . . To go to a mental health practitioner and have them say “oh you seem pretty fine,” I feel that that experience can almost amplify the negative self-talk like “see it’s all in your head” or “you’re the problem,” “you’re the only one who thinks there’s a problem,” “it’s not true,” “why do you think you’re so special,” all those things. (YLER member)

## Avoiding objectification

### An agent may have multiple and conflicting needs and interests

People are *objectified* when their status as subjects of experience and agents with a multiplicity of goals and interests is not taken into account. In the mental healthcare context, young people are objectified when they are seen as a category of patient or the embodiment of a specific problem to solve. For example, in the video data, objectification occurs when a practitioner tells a young person they need to attend talking therapy “because that’s what works for depression” without accounting for competing needs, fears, or experiences that may feel important to the young person (Eldal et al., 2019; Hartley et al., 2022).

Martha Nussbaum (1995) lists several ways in which objectification can manifest in relationships, and her discussion suggests that objectification is inconsistent with the agential stance. For instance, objectification is incompatible with validation when “the objectifier treats the object as something whose experiences and feelings (if any) need not be taken into account” (p. 257). Another manifestation of objectification is *fungibility*: “the objectifier treats the object as interchangeable with other objects of the same type, or with objects of other types” (p. 257).

A common effect of objectification is *labelling* someone, or putting someone into a box. People are labelled when they are assigned a category. For example, labels may be about social status, physical appearance, or moral attributes. The labelling is detrimental when it has the effect of trivialising the person’s identity and constraining the person’s opportunities for growth and action. In the mental healthcare context, the most salient type of labelling involves practitioners redescribing the young person’s concerns so that they fit a certain diagnostic category or considering the young person’s concerns merely in the light of risk assessment (McWilliams, 2011).

A diagnosis can bring relief and justification to a young person as to the symptoms they have been experiencing regarding their mental health, but it can also cause them to conflate their illness as their identity especially in their vulnerable state. By internalising their diagnosis, they may start associating that as their general character when that should not be the case. Hence why practitioners need to be more tactful when delivering a diagnosis to not make the young person feel they are anchored to their mental health issues. (YLER member, written memo)

When you’re a young person, your identity is so malleable. The moment you get a diagnosis, it’s very easy for it to become enmeshed with your sense of identity (be it in a positive or negative way). (YLER member)

While an appropriate diagnostic label can offer support and relief, premature or superficial labelling causes lasting damage. It is particularly problematic when the young person’s concerns are inappropriately or preemptively labelled before they are fully explored. The YLER describe feeling both objectified and prematurely labelled when practitioners propose a diagnosis without inquiring about other psychological or social factors that may feel important to that person (e.g., family, school):

Life is hard. And instead of them dealing with it in a more holistic way, they just, they’ve just been put into this box. Oh this is what I have. . . It’s like a simple explanation for something that has many complexities. And I feel like that’s the thing about seeking medical attention is they just give you a name. (YLER member)

Premature or superficial labelling and objectification may be seen as a means of legitimising the young person’s choice to seek help; if there is a legitimate concern that can be met by mental healthcare professionals, then the practitioner can give it a name. However, the YLER stressed that premature and superficial labelling and objectification actually delegitimised their concerns and made them feel it was justified for them to seek help only in so far as their concerns conformed to the practitioner’s expectations.

An example of objectification potentially leading to premature labelling is provided in Extract 5. The young person was brought to the emergency department by the university after telling a classmate that they felt suicidal.

Extract 5

1 Pr: What- What kind of plans would you have had [tonight]?

2 YP: I- It was- I’d got a few events on. Because I’m part of

3 rugby, skiing and tennis and they all had different events

4 on tonight like every Monday.

5 Pr: I see. So w- we could safely say you’re not going to end

6 your life? Do something that would have-

7 YP: Not tonight.

8 Pr: Yeah. Yeah. It-

9 YP: I wouldn’t have ended it tonight, no.

10 Pr: Okay. So maybe there was a bit of miscommunication. ‘Cause

11 they brought you here ‘cause they were saying that you

12 were suicidal and-

13 YP: No I am. But like I’ve got-

14 Pr: You are?

15 YP: I’ve got- I’ve- I feel I can- I mean I haven’t done it yet.

16 Pr: Mm. Mm.

Just after the young person confirms that they did not have immediate plans to end their life (“not tonight,” lines 7/9), the practitioner proposes that the university miscommunicated by stating that they were suicidal (lines 10–12). Instead of validating the young person’s complex experience and exploring their thoughts and intentions, the practitioner labels them “nonsuicidal.” Given that this is a crisis support service, the label also effectively delegitimises the young person’s concerns. The young person immediately counters the label and struggles with their words as they go on to try to explain their experience (lines 13/15). The practitioner focuses on whether the young person is suicidal and treats this as a question with a clear “yes or no” answer, assuming that the young person having plans for the evening is incompatible with the label “suicidal.” This relates to another important aspect raised by YLER, namely what being suicidal looks like and whether a person can look or act in a way that does not fit others’ ideas of what suicidal looks like.

In Extract 6, a young person speaks with the practitioner and describes their experiences with superficial labelling:

Extract 6

1 YP: I’ve always been told that it’s just anxiety and depression.

2 Pr: Mm. What’s your point- What do you think yourself?

3 YP: I don’t know. I just feel like that’s- kind of- I’m not sure, not a

4 cop out, but I feel like they haven’t- doctors that I’ve spoken to

5 haven’t really bothered to like go deeper. And they’ve just thought

6 “She’s a young person. I’m gonna look at these symptoms. Ignore the

7 rest. And just fit it to what I want it to be.”

8 Pr: I see. I see.

9 YP: So I feel like I haven’t ever had the right treatment really. But I

10 don’t know why. But I just don’t feel like I have.

Like the young person in this extract, the YLER emphasised the long-term negative effects that objectification and premature or superficial labelling can have on treatment and the health trajectory. These include ill-fitted treatment plans, mistrust of services, hesitancy to open up to others (e.g., about thoughts of suicide), and losing a sense of identity. While objectification has negative consequences, the YLER agreed that appropriate labelling following substantive exploration, validation, and legitimisation is largely beneficial. Appropriate diagnosis, especially, can be liberating as it has the potential to link the young person to communities that understand what the young person is experiencing and to treatment programmes that can provide the right sort of support:

I feel that labels can be assigned too early before proper investigation of the young person’s experience, which leads to inappropriate treatment being provided which can cause more harm than good. For example, in my case, I have been given psychotherapy and CBT to help with my mental health difficulties, but I didn’t feel any benefit and it actually caused greater anxiety. . . I am now being investigated for an ASD diagnosis and they’re not surprised that I was unresponsive to previous therapies as it often doesn’t work for those with ASD and I am now [benefitting from] alternative therapy, specifically music therapy. (YLER member, written memo)

## Affirming the young person’s capacity to contribute to change

### With adequate support, an agent can contribute to positive change

In philosophy of mind and action, when we consider someone as an agent, we consider them as someone who can be (at least partially) responsible for their actions and choices because their actions and choices reflect their needs and interests (e.g., Bratman, 1987; Davidson, 1980). In the psychotherapy literature, agency is attributed to clients who “make and enact choices regarding their therapy” and enables clients to participate actively in therapy, acquire and share knowledge, value their accomplishments, and feel empowered (Hoener et al., 2012, p. 66).

Responsibility and choice are central features of agency: competent agents capable of deliberation and planning are (at least partially) responsible for their past and future actions and choices. On such bases, they can shape their lives and contribute to their identity. However, in the mental healthcare context, the concept of responsibility can become problematic. Assigning responsibility is linked to praising or blaming. According to Hanna Pickard (2011), this gives rise to a problem for mental healthcare practitioners:

[The] requirement of effective treatment creates a clinical conundrum. How is it possible to hold service users responsible for behavior that causes harm and suffering, to the self and, especially, to others, without blaming them for it? Encouraging responsibility is central to effective treatment. Blame, in contrast, is highly detrimental. (p. 210)

Pickard proposes the responsibility without blame framework for the relationship between practitioner and user. Crucially, she maintains that attributions of responsibility can be detached from praise or blame. Whereas responsibility indicates that the agent intentionally initiated that action or choice, judgements of praiseworthiness or blameworthiness depend on the level of control that the agent had over their options and the extent to which they could have acted or chosen differently.

Both theoretically and practically, though, detaching responsibility from blame can become a challenge and is not always desirable because some of the motivation that agents have for implementing positive change may be due to a desire for praise rather than a realistic estimation of the role they played in the situations they wish to avoid. That is precisely why Brandenburg and Strijbos (2020) endorse an alternative framework, called the *nurturing stance*. In the nurturing stance, blaming attitudes are interpreted as guidance for the future rather than an attempt to judge the agent for their past: “The nurturing stance begins from the assumption that the person is not yet (fully) responsible but can be supported to become so. The responsibility of the service user is thus primarily forward-looking, not backward looking” (Kennett, 2020, p. 397). The nurturing stance could be especially helpful in the context of mental healthcare users who are young people, as their capacity to assume responsibility for complex actions and choices may be affected by their limited life experiences and early developmental stage. Attributions of responsibility should not become a burden for the young person and should not imply that support is no longer needed: “Calling upon the person to have a self-governing approach does not imply, of course, that the service user will not be offered the help and support needed” (Brandenburg & Strijbos 2020, p. 386).

In the analysis of video-recorded psychosocial assessments, the YLER observed that it was difficult for practitioners to communicate that the young person had the capacity to assume responsibility without implying that the young person did not need support or without blaming them for past behaviours. In contrast, the YLER identified more positive patient responses when the practitioner affirmed the young person’s capacity to contribute to change. Reflecting on the philosophical literature introduced above, the YLER emphasised the importance of the distinction between the young person’s capacity to assume responsibility (which places a burden on the young person) and the young person’s capacity to contribute to change (which is compatible with the young person’s behaviour being just one of many relevant factors in their mental health journey).

With respect to what the young person has already experienced, this involves recognising and affirming the positive work the young person has already done to get where they are and to manage the crisis they are in. For example, making it through each day, seeking help from others, disclosing their mental health concerns, and practising coping strategies at home. With respect to what the young person will experience, it involves recognising the hard work the young person will continue to do in the future, affirming their goals of engaging with further support and taking steps forward. For example, the goals of attending talk therapy, trying new coping strategies, asking for support when things get worse, or simply continuing to make it through each day.

Practitioners should acknowledge that the young person’s current state of distress is not their fault or a direct result of their actions. But they should also encourage the young person and note that they have the inner strength and capacity to eventually redirect and change their current circumstances (almost like a scaffolding approach). (YLER member, written memo)

For the practitioner, affirming the young person’s past contributions to change and their capacity to contribute to future change promotes a recognition of the young person as a capable agent. This means that practitioner and young person have a shared understanding of the difficult work the young person is already undertaking and will be undertaking in the future to manage their health.

In Extract 7 the young person had overdosed many times in the previous year, but then started attending therapy and stopped overdosing as frequently. They presented to the ED following their first overdose in many months and were seen by two practitioners.

Extract 7A

1 Pr1: It’s really hard to change things. When you’ve got coping

2 strategies or whatever it is it’s really really hard to change it.

3 And you’ve done really well to change. I saw you last year. I saw

4 you a few times last year.

5 YP: Yes.

6 Pr1: When you talk about your bad times I- that’s how I remember you.

7 That’s how I met you. And things are completely different for you

8 now.

9 YP: Yeah. Yeah. I am doing better.

10 Pr1: I think you should be really proud of what you’ve done. A lot of

11 people don’t manage to do it. I think you’ve done really really

12 well.

Extract 7B

20 Pr2: And the best predictor of you know- of what’s possible for the

21 future is what you’ve managed- what you’ve achieved.

22 Pr1: Yeah.

23 Pr2: So you’ve had really- this really big chunk of positive time.

24 Which means that [gestures] you can expand that going forward.

25 YP: [nodding]

26 Pr1: Absolutely.

In lines 1–2, the practitioner communicates a message that strongly resonated with the YLER: “When you’ve got coping strategies [e.g., self-harm] or whatever it is it’s really really hard to change it.” The YLER stressed the importance of conveying this message to young people that present with self-harm behaviours (cutting, binge drinking, overdosing, etc.). The practitioners acknowledge the young person’s past achievements (Extract 7A). They then link these to an affirmation of the young person’s ability to contribute to further positive change in the future (Extract 7B).

Affirming the young person’s capacity to contribute to change necessarily builds on validation and legitimisation. Without communicating to the young person that they are understood and they are deserving of further support, affirmation of their capacity to change could problematically imply that the young person should be able to manage on their own without additional support.

The YLER also emphasised the damaging effect of implying that negative outcomes (e.g., family members’ distress, health problems) were a direct result of the young person’s actions. In our observations of encounters in the mental healthcare setting, practitioners often blame young people for self-harm. Blame does not take the form of a nurturing reproach aimed at the future but instead is applied to past actions, potentially triggering a sense of guilt and negatively affecting the young person’s conception of themselves.

[Please note that the following transcript contains references to suicide.]

Extract 8 provides an example of a practitioner blaming a young person on both epistemic and moral grounds. The young person was found by their sister and brought to the hospital following an attempted suicide by overdose.

Extract 8

1 Pr: So when you say you’re concerned about the impact on other people.

2 Had you thought about s- how- what would happen: for the people

3 that found you?

4 YP: I- I had. I didn’t want it to be my sister.

5 Pr: Okay? … But you said she was in the house at the time. So it

6 could have been her.

7 YP: It could have been her. Yeah.

8 [long silence]

The practitioner cites the young person’s earlier statement that they were concerned about the impact that their overdose might have on the people around them (line 1). The practitioner then immediately asks whether the young person had thought about what would happen to the person who found them after the overdose (line 2). By asking the question here, the practitioner implies that the young person was not showing concern for the person who would find them. After the young person confirms that they had thought about this and they had not wanted their sister to find them (line 4), the practitioner points out that they earlier stated their sister was in the house at the time (line 5), implying that the they knew that there was a possibility that their sister would find them.

Extract 8 shows instances of a young person being blamed both epistemically and morally. Agents can be blamed on epistemic grounds if they are thought to fail to gain or use relevant knowledge in the appropriate way, with undesirable consequences. In the crisis care setting, practitioners epistemically blame a young person when, for instance, they indicate that the young person should have considered the medical risks of self-harm or should have known the consequences of not taking the medication prescribed to them for a mood disorder. These attributions of blame by practitioners also assume that the young person was in complete control of their mental states and actions, and that past practitioners clearly communicated the risks to the young person—not all GPs convey the risks of not taking medication or adequately prepare their patients for how difficult the first few weeks of medication can be.

The practitioner who attributes blame fails to consider the context of the young person’s choices, actions, or omissions. For example, instead of inquiring about a young person’s reasons for not taking their medication (e.g., side effects, unstable living environment, etc.) and taking those into account, the practitioner simply condemns the young person’s behaviour. The YLER argued that by taking this approach, the practitioner loses out on obtaining vital information that they could use to help the young person achieve any medication adherence goals they might have. If the young person feels ashamed, they may be less inclined to disclose the reasons why they were unable to adhere to their treatment plan.

Agents can also be blamed on moral grounds if they are thought to act in a morally objectionable way, with adverse consequences for themselves or others. In the crisis care setting, practitioners attribute moral responsibility in a way that imposes blame when, as we saw in Extract 8, they suggest that a young person who overdosed should have considered the negative impact of attempting suicide on their family and friends. This approach has the added consequence of invalidating the young person’s distress, because the practitioner implies that the distress caused to the young person’s family and friends is of greater importance than the distress that the young person is trying to escape from. The YLER suggested that the message is: “It is not relevant how you’re feeling. Just imagine what this would do to everyone around you.”

## Involving the young person in the decision-making process

### With adequate support, an agent can participate in decision making

One important aspect of recognising someone as an agent is to enable their participation in decision-making processes that bring about change (Larner, 1998) and value their contribution to shared projects. In the mental health context, the young person may be asked to express their point of view on potential sources of support, such as bereavement therapy or peer support groups, based on their previous experiences (see Barnes, 2018; Bergen & McCabe, 2021).

Involving the young person in decision making implies a rejection of objectification and an endorsement of their capacity to contribute to change. We can regard the people we speak to as either informants or sources of information (Fricker, 2007). If we regard them as mere sources of information we imply that they do not actively participate in the process of producing and sharing knowledge. When we consider them as informants, we involve them in that process and value their active participation. The idea that paternalistic approaches to medicine should be left behind and the person using healthcare services should be viewed as a partner participating in a collaborative effort has been promoted widely within physical (e.g., Coulter, 1999; Karazivan et al., 2015; Shay, 2014) and mental healthcare (Priebe et al., 2011). But for the collaboration to work, the practitioner needs to recognise the person using healthcare services as an agent who can produce and share knowledge and also contribute to change (Munthe et al., 2012). Video analysis for the present study revealed limited involvement of young people in treatment decisions.

Extract 9 provides an example of a disempowering treatment conversation in which the young person is not offered additional support and is excluded from the decision-making process. In this case, the young person presents with suicidal thoughts. They were previously prescribed antidepressants but stopped taking them after experiencing some side effects.

Extract 9

1 Pr: Do you want to try and address your mood?

2 YP: What do you mean?

3 Pr: Do you want it to be different from what it is?

4 YP: Yeah.

5 Pr: Yeah?

6 [silence]

7 Pr: So what do you need to start doing?

8 [silence]

9 YP: Taking tablets.

10 Pr: Yeah. Because what dose did you say you thought it was?

11 [silence]

12 YP: Twenty milligram.

13 Pr: Twenty. You need to g- give it a go Jack.

The practitioner asks the young person to identify what they need to do in order to change their mood (line 7), implying that they already have all the support they need to improve the situation. After a long silence, the young person answers the question (line 9) but does not indicate that they plan to take the antidepressants. At no point in this treatment conversation does the practitioner ask for the young person’s perspective on the treatment, help them set realistic goals, or involve them in the treatment decision-making process. This means that the young person does not have the opportunity to collaborate with the practitioner in identifying appropriate support that they feel able and willing to commit to.

The YLER stressed the importance of involving young people in treatment decision making for long-term outcomes:

If the young person and practitioner don’t have an open discussion about goals for treatment and the young person’s preferences, they may end up working against each other throughout the duration of treatment and may make little to no progress. It becomes a battle/tense interaction of “who knows more,” instead of what should be an active collaboration of sharing knowledge and perspectives to facilitate the young person’s recovery. (YLER member, written memo)

It’s crucial that a young person is given the opportunity to identify appropriate treatment for themselves. A practitioner’s role is to communicate what the best solution will be based on their specialised knowledge, but it should be up to the young person to absorb that knowledge and choose a course of treatment that they see will best improve their life. Instead of it being enforced to take medication/therapy they should feel empowered to take responsibility as it demonstrates that they have the intelligence and agency to get better. (YLER member)

Extract 10 involves an empowering treatment conversation in which the young person is asked to share their perspective on a possible source of support before the practitioner makes a recommendation. The young person recently lost their mother and presented to the emergency services following an overdose.

Extract 10

1 Pr: On the subject of your- of your mom have you: ever had any

2 counselling. Any grief counselling or |around

3 YP: |No.

4 Pr: loss of a |family member. No.

5 YP: |No.

6 (4.0)

7 Pr: Has anyone ever suggested it to you.

8 YP: The mental health team did in London. They suggested counselling

9 for |me, uh

10 Pr: |Okay?

11 (1.0)

12 Pr: What do you think about that.

13 (2.0)

14 YP: hhhhh It’s just talking about it I just don’t wanna keep do:ing,

15 But I- you know, it upsets me. I don’t want to feel upset. I don’t

16 wanna talk about mum.

17 Pr: Okay.

Before recommending grief counselling, the practitioner asks if the young person has had this form of counselling in the past. When the young person doesn’t immediately disclose their perspective on grief counselling (lines 3, 5, 8–9), the practitioner asks specifically what the young person thinks about that form of support (line 12). The young person is able to clearly explain the reasons for their hesitancy to engage with this type of treatment (lines 14–16). As a result, the practitioner ultimately recommends a different type of treatment, namely cognitive behavioural therapy (CBT). The practitioner does so specifically referencing this earlier conversation and framing CBT as a form of therapy that doesn’t focus on past trauma but rather helps to develop coping strategies.

The YLER described involvement in decision making as an example of collaboration in which both the young person’s and the practitioner’s perspectives are valued. If the practitioner’s perspective is missing, the treatment decision is a burden to the young person. If the young person’s perspective is missing, the treatment decision is disempowering:

It’s important to recognise that considering the young person’s preferences doesn’t mean that they will direct every aspect of the treatment. . . Rather, it means acknowledging that both the young person and the practitioner have valuable information to bring to the table and to inform the young person’s treatment. For the young person, it may be being an expert of their own life – their past (positive or negative) experiences with different treatment approaches, their own insight in their capacity to challenge themselves or cope with certain demands of treatment and their general preferences. For the practitioner, it could be years of clinical experience. (YLER member, written memo)

Incorporating the young person’s “voice” in their treatment does not mean invalidating the clinical competency of the practitioner. Similarly, acknowledging the inherent power imbalance/help-providing nature of the practitioner does not dismiss the validity or usefulness of the young person’s beliefs. (YLER member)

## Conclusions and implications

In this article, we identified communication techniques that practitioners can use to protect young people’s sense of agency in the mental healthcare context. Although our analysis focuses on five communication techniques, we expect that further research will reveal other useful practices that prevent practitioners from inadvertently undermining young people’s sense of agency in the context of a clinical encounter.

Our study focuses on young people who approach mental healthcare service during a crisis. However, the practices we described may also apply to different clinical populations and, more widely, to personal relationships with a power imbalance (e.g., parent and child; teacher and student; employer and employee). The power imbalance may turn on the dominant party having authoritative knowledge or greater experience, being in a position to advise the subordinate party, or being the gateway to support, such as access to services.

**Figure 1.**
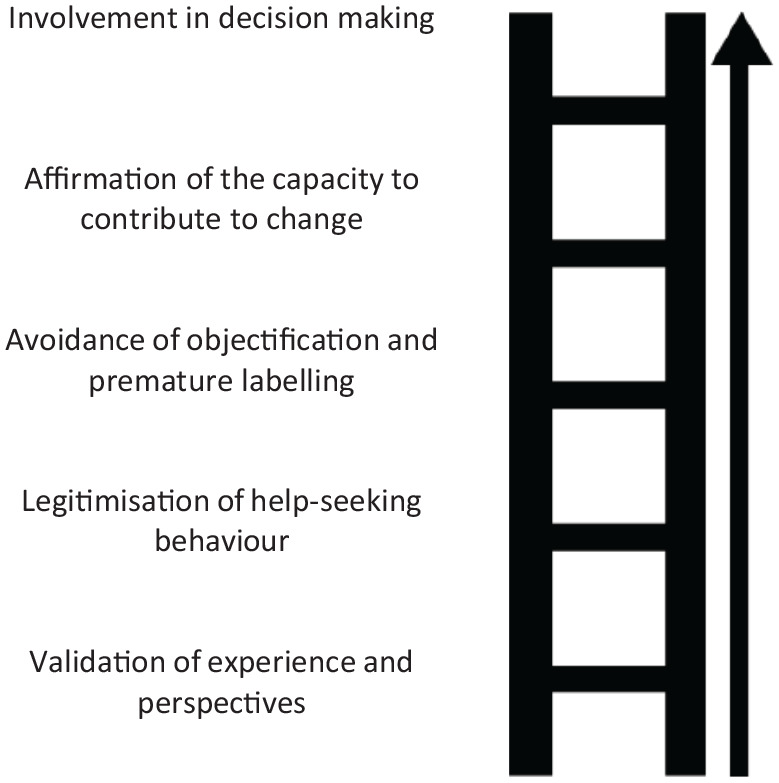
The agential ladder.

We can visualise the agential stance as a ladder, with each step contributing to the practitioner protecting the young person’s sense of agency (see Figure 1).

The agential ladder is a tool we propose for empowering young people in clinical encounters and for gaining a better understanding of why certain communication practices have a positive impact on young people seeking support. We hope that the agential ladder will invite some reflections on the pivotal role of effective communication in empowering young people without overburdening them. Whereas valuing the young person’s experiences and affirming their capacity to contribute to positive change are likely to have beneficial effects on the therapeutic relationship and on health outcomes, it is problematic to attribute excessive responsibility to the young person at a critical time or ask them to make decisions without adequate support.

The agential ladder should not be seen as an overly demanding constraint on mental healthcare practitioners. Ensuring validation and legitimising concerns are already significant steps in the right direction. Validation is instrumental to the quality of the therapeutic relationship, which is what determines whether young people remain engaged in mental health care and predicts mental healthcare outcomes (Priebe & McCabe, 2006; Ryan et al., 2021). Validation enables judicious diagnoses and risk assessments, and informs the decision-making process surrounding treatment options and further support.

The practices of validation and legitimisation as described by the YLER evoke key moral and epistemic virtues, such as compassion and intellectual curiosity. These manifest in listening attentively and empathetically, and asking meaningful questions to further explore the other person’s experience:

I think in initial interactions, there needs to be a strong theme of compassion and curiosity from the practitioner. There should be a strong sense of compassion towards the young person’s difficulties and circumstances (both within and beyond the consultation room) that are impacting [their] sense of agency and capacity to take charge of [their] mental wellbeing. This can be conveyed through validating statements and “holding space” for the young person. There should also be a sense of curiosity from the practitioner – they should truly try understand why the young person is in their current state of distress and how the young person feels about their circumstances (rather than just going straight into telling the young person what they should/shouldn’t do or how their current actions are wrong). This fundamentally not only preserves the young person’s sense of agency, but also helps the practitioner gain a more comprehensive, rich understanding of the young person’s circumstances – which could be used to maximise the effectiveness of future management/care plans for the young person. (YLER member, written memo)

Validation is a critical tool that benefits both the young person and the practitioner:

–It allows the young person to feel safe and understood, and to have an overall positive experience with services (which would encourage future help seeking)

–It also gives the practitioner more rich information for their assessment notes.

This can help inform the specific care they provide for the young person (which in turn could make interventions more effective and reduce repeated ED admissions, since practitioners are understanding the underlying perpetuating/contributing factors to the young person’s problem very early on). (YLER member, written memo)

Despite the systemic structures that limit the practitioner’s ability to conduct comprehensive, holistic, and person-centred safety assessments, communicating validation and legitimisation does not require a considerable investment in time or resources. As observed in the recordings, it can simply amount to saying: “That makes sense.”; “That’s a really difficult experience.”; or “It was the right decision to seek help.” (see Table 1). The young people interviewed as part of our study were aware of the strain experienced by mental health services and emphasised that validating and legitimising practices would go a long way in changing their perceptions of the clinical encounter for the better.

**Table 1.** Key aspects of agency and communication techniques with examples from video data.

Aspects of agency	Goals	Examples of communication techniques
An agent is a subject of experience and their perspective matters.	Validate	Treat the person’s feelings as valid: “It’s a really horrendous event.” “That’s a scary thought.” Treat the person’s story as important: “Thank you for being so open and honest about these things.”
An agent can take action to change their situation by seeking help.	Legitimise help seeking	Commend the person for seeking help: “Well done. . . you did exactly the right thing.” Encourage future help seeking: “Our message to you today is that it’s okay to talk to people about these things and it’s very important that you do.”
An agent may have multiple and conflicting needs and interests.	Refrain from objectification	Acknowledge a multiplicity of factors contributing to the mental health crisis: “There’s lots of things we’ve already talked about that are contributing to you feeling low at the moment.” Ask what may have been missed: “Is there anything else we haven’t talked about you think is important?”
With adequate support, an agent can contribute to positive change.	Affirm capacity to contribute to change	Acknowledge changes they’ve already made: “I think you should be really proud of what you’ve done.” Emphasise teamwork: “It’s about enabling you or supporting you to develop strategies and skills. . . And that’s what the [mental health team] will be doing.”
With adequate support, an agent can participate in decision making.	Involve in decision making	Ask for the person’s perspective on treatment: “What do you think about that [treatment option]?” Ask about past experiences with treatment: “What about the [previous treatment] was most helpful?” Provide an overview of the options: “Different things are right for different people. . .”

In sum, by adopting the agential stance, practitioners take steps to protect the young person’s sense of agency at a time when it is likely to be threatened by a mental health crisis: arguably, this enhances the quality of the therapeutic relationship and has potential to indirectly improve clinical outcomes. In the light of this, our recommendation is that the risks of undermining young people’s sense of agency be discussed in the training offered to mental healthcare professionals, with examples of good and bad practice clearly presented to trainees.
